# Consumer Insights into “Clean Label” High-Fat, Low-Carbohydrate Protein Bars

**DOI:** 10.3390/foods15030551

**Published:** 2026-02-04

**Authors:** Meghan M. Stewart, Md Shakir Moazzem, Jordan N. Proctor, William L. Kerr, Mackenzie J. Bui, Koushik Adhikari

**Affiliations:** 1 Department of Food Science and Technology, University of Georgia, Athens, GA 30602, USA; meghan.stewart@uga.edu (M.M.S.); wlkerr@uga.edu (W.L.K.); mackenzie.bui@uga.edu (M.J.B.); 2 Department of Food Science and Technology, University of Georgia, Griffin, GA 30223, USA; mdshakir.moazzem@uga.edu; 3 Department of Animal & Dairy Sciences, University of Georgia, Athens, GA 30602, USA; jordan.proctor@gmail.com

**Keywords:** acceptability, snack bar, meal replacement bar, protein bar, meat bar, meat snack product

## Abstract

This study assessed consumer perceptions of high-fat, low-carbohydrate (HFLC) protein bars containing varying levels of beef tallow fat. A consumer acceptability test was conducted (n = 102) with four prepared and one commercially available HFLC bar samples. Hedonic, diagnostic (intensity), and just-about-right (JAR) questions on overall liking, texture, flavor, and purchase intent were included in the sample evaluation ballot, followed by general demographic, consumption behavior, and ingredient preference questions about the product category. Although none of the samples, including the commercial bar, were liked, the sample with the highest protein content and lowest fat content was preferred over the others. Overall flavor and aroma liking were rated significantly higher for all prepared samples compared with the commercial bar (*p* ≤ 0.05). The sample evaluation revealed potential pathways for improving HFLC bars by leveraging “fat-synergizing” attributes such as sweetness, saltiness, and spiciness, with texture improvements possible through higher lean-protein incorporation. The ingredient factors most important to the participants were high protein content, high satiety, minimal ingredients, natural ingredients, and no added sugar. This study’s results demonstrate a widespread desire for fewer ingredients overall, more natural ingredients, and high satiation in snack products.

## 1. Introduction

In the current food industry landscape, with regulatory changes and growing consumer demand for “clean label” food products, food scientists need to respond quickly to the call to reformulate conventional products. While there is no legal or standardized definition of clean-label foods, several interpretations have emerged from market and consumer influences. According to Asioli et al. [1], three broad categories drive clean-label trends: “organic,” “natural,” and “free from artificial additives/ingredients.” Perceptions of how these factors manifest in food products are greatly influenced by consumer psychology, sociocultural factors, media and information exposure, regulatory knowledge of food additives, and intrinsic product characteristics, such as the presence of additives, food product associations with a given ingredient, and the degree of processing [1]. The Nova food classification system, which breaks down foods into categories based on the level of processing, defines ultra-processed foods (UPFs) as foods containing more than five ingredients, particularly ones not commonly found in a household kitchen, such as artificial colors, flavorings, preservatives, and other compounds used for increasing palatability, shelf life, or cheapness to produce [2]. A Glanbia Nutritionals survey [3] found that 53% of surveyed consumers were concerned about UPFs, with the concern rising to 71% among “health-conscious” consumers, and that 14% of all consumers said they eat fewer snack bars because of this [3]. At the same time, health and fitness have become a top priority for consumers worldwide, leading to increased demand for protein bars and energy-boosting snacks. The United States snack bar market is estimated at $11 billion to $13.2 billion in 2024, accounting for 39–43% of the total global market. Energy and nutrition bars dominate the space, accounting for 66.2% of total snack bar revenue in 2024. Key growth drivers include high-protein demand, fitness culture, and the convenience of meal replacements [4]. The 2025 forecast for the global protein bar market size is currently calculated at USD 15.26 billion, accelerating at a CAGR (compound annual growth rate) of 5.1% from 2025 to 2034. Sports nutritional bars accounted for the highest revenue share in 2024 at 51%, and animal-based protein captured the most significant share by protein source at 77% [5]. Approximately 68% of polled American consumers are interested in healthier snack options, and demand for protein-enhanced snacks has increased by 30%. Demand for convenience has led many consumers to use snacks as meal replacements [4]. Meal replacement bars grew at a CAGR of 6.3% over the 2025–2034 forecast period, with portable convenience foods helping to form the market for meal replacements among the market-dominating travelers and on-the-go professionals. Clean-label consumer preferences are now manifesting as a desire for products containing natural, familiar ingredients without artificial additives, added sugar, or preservatives, and with minimal ingredient lists [5].

Beyond shelf life and ingredient costs, the profitability of clean-label food products is affected by consumer preconceptions about the palatability of “healthy” snacks and the formulation challenges of using limited “clean label” ingredients. Legacy protein bar brands continue acquiring new clean-label brands to expedite adaptation to consumer avoidance of mainstream product offerings now perceived as “ultra-processed foods.” Energy bars that successfully deliver dessert-like taste while being high in protein and low in sugar are growing the fastest in this current market landscape, while other dietary segments, such as plant-based/vegan and ketogenic/low-carbohydrate, are not-so-quietly influencing the entire innovation space and emerging formulation trends [4]. The decline in plant-based innovation in 2025 reflects a significant disconnect between industry and consumer visions of “clean label” [6], underscoring the need for food scientists to investigate other trending diets to reformulate traditional UPFs with a consumer-informed approach. Meat snack products, however, face unique challenges in the clean-label innovation space due to the ubiquitous use of functional additives for preservation and texture enhancement, where few natural alternatives exist to nitrates, phosphates, and texture stabilizers [7]. Creativity in research and development is required to use ingredients such as herbs, essential oils, and spices, along with minimal processing techniques, to develop clean-label meat products [7].

Ketogenic snack bars focus on moderate protein, mainly high-fat “whole-food fats,” and have low carbohydrate content, with an emphasis on natural sweeteners. In the broader snack bar market, ketogenic diets have significantly increased demand for low-sugar and low-carbohydrate products, while aligning with the general population’s growing desire for whole-food-based snack products [4].

While low-fat food trends continue to shape global food choices, there has been a recent increase in fat intake, particularly in whole-food animal and plant fats. Among those surveyed in the Nielsen Company industry report [8] who aimed to lose weight, the use of high-fat, low-carbohydrate diets increased by 34% in 2014. These diets are characterized by a focus on animal proteins and fats, low-sugar fruits and vegetables, limited whole grains and added sugars, and unprocessed “natural” foods. Health beliefs, including perceptions of a food’s healthiness, play a key role in driving consumer behavior. High-fat, low-carbohydrate (HFLC) dieters perceive whole grains, sugars, refined wheat products, and vegetable oils as harmful to health, compared with other groups, and saturated fat—particularly animal fats—as more beneficial to health [9]. Ketogenic and paleo diet principles have heavily shaped changing views of “clean label,” now emphasizing the removal of undesirable, non-natural ingredients and sugar while prioritizing minimal processing and whole-food nutrition [10]. Consumer preferences for products in clean-label diet categories, such as ketogenic and paleo food products, can be used to gauge the potential usefulness and desirability of these formulation formats for the broader clean-label snack bar innovation category, due to their nature of showcasing whole-food, minimally processed ingredient lists which align with more generalized “clean label” consumer perceptions [1,11]. One keto/paleo product type is emerging in the clean-label snack bar market as a champion of minimally processed, whole-food ingredient products: the pemmican bar. With ancient indigenous North American origins and a high nutrient density, this original “protein bar” used a mixture of desiccated dried meat, animal fat such as suet, and, often, dried fruit. Pemmican was historically relied on as a core food item with higher satiety and palatability than jerky for soldier rations, arctic explorers, and fur traders alike. Historic documentation on its preparation is scant and reveals wide variability in formulation, with fat–dried beef mass ratios ranging from 1:1 to 4:5. Pemmican’s vital role in population sustenance led to the Pemmican Wars (1812–1821) over its rationing in North American history [12].

Previous studies and U.S. Department of Defense efforts have examined the suitability of pemmican as a military ration, given its historical use as a portable, nutrient-rich survival food for extreme environmental conditions. However, they have so far failed to explore the use of pemmican or other HFLC rations in temperate climates due to past issues with palatability and acceptability [13,14,15]. Renewed interest in its use is only beginning to be explored in current research, with a focus on performance benefits and nutrition, but not on sensory appeal [16,17], despite commercial launches of pemmican-style snack bars and meal replacement bars under clean-label or paleo/ketogenic marketing Although consumer acceptance of meat snack bars similar to pemmican has been assessed [18,19,20,21,22,23], research on the palatability and sensory appeal of traditional North American pemmican formulations for modern-day consumers is lacking. For these reasons, the current investigation was conducted to assess the modern consumer’s acceptance of pemmican snack bars and the potential for mainstream appeal of commercially available pemmican snack bars that showcase “clean label” processing and ingredient label attributes.

The overall objective of this study was to understand consumers’ sensory perception of high-fat, low-carbohydrate (HFLC) protein bars. The specific objectives included assessing overall consumer acceptability of “pemmican”-style protein bars with different fat levels, examining consumers’ flavor and texture perceptions of HFLC protein bars as fat content increases, and exploring consumer preferences and attitudes toward “clean label” ingredients. To achieve these goals, a consumer test was conducted to assess the acceptability of five HFLC protein bars formulated as “pemmican”-style bars with varying fat content, including a commercially available pemmican bar for comparison and benchmarking. Additionally, participants’ attitudes toward “clean label” ingredient factors when purchasing snack bars, protein bars, or meal replacement bars were examined.

## 2. Materials and Methods

### 2.1. Materials

Top-round beef, sliced into slabs less than 1.27 cm thick against the grain, was purchased from Publix © Super Markets, Inc. (Lakeland, FL, USA), as well as black pepper (Badia Spices© LLC, Doral, FL, USA) and garlic powder (Badia Spices©). Other ingredients purchased online included 100% suet-derived, grass-fed beef tallow (The Fat Lady Tallow©, Palmyra, WI, USA), dried mango (Anna and Sarah©, Somerset, NJ, USA), habanero flakes (Sonoran Spice©, Scottsdale, AZ, USA), ground white pepper (Felicific Inc., Queens, NY, USA), and sea salt (Sosalt Spa, Trapani, Italy). A market product in honey barbecue flavor, referred to here as the commercial bar (Carnivore Bar©, Columbia, MO, USA), was purchased to serve as a 5th sample during consumer panel testing. This commercially available bar was included to compare and benchmark against the prepared samples, given its market success, exemplary demonstration of pemmican bar ingredients in food product development, and comparable fat content to the highest-fat prepared sample. The chosen flavor, honey barbecue, was the most similar to the prepared sample among commercially available flavors, while offering a contrasting flavor for acceptability comparisons at the highest fat content level.

### 2.2. Sample Preparation

Four samples of the same flavor (mango habanero) were developed using equal percentages (*w*/*w*) of spices. The ratio (*w*/*w*) of beef tallow to beef protein was adjusted to create four macronutrient variants, with estimated calories from beef protein representing 21%, 24%, 27%, and 30% of the total calories. A single-serving formula of 15 g of dried beef and 7.5 g of mango was used for each bar, while the beef tallow volume was increased incrementally to develop batch recipes (Table 1). Referenced protein percentages were used for sample labeling purposes only and calculated assuming near-100% protein content from the desiccated beef, approximately 4 kilocalories (kcal) from protein, and 9 kcal from fat [24]; thus, these percentages do not reflect the final protein content relative to fat. Samples were prepared by slicing the purchased top-round beef into 5 cm wide strips, trimming away the fat, and marinating in a salt brine (3.75% brine concentration) for 24 h to facilitate dehydration. The beef was then dehydrated on bacon hooks without smoke in a smokehouse following a 4-step dehydration schedule. Step 1 utilized steam heat and a dry-bulb (DB) temperature of 52 °C for 3 h, followed by 1 h of heating at step 2 at 60 °C at 55% relative humidity (RH), followed by step 3, which involved heating the beef to 74 °C at 40% RH for 30 min. The fourth step was run at 100% relative humidity, with no applied heat, for 72 h. After removing from the smokehouse, eight randomly selected pieces of dehydrated beef were measured for water activity (Aw) using an Aqualab^®^ Series 3 water activity meter (Decagon Devices, Inc., Pullman, WA, USA). After a target water activity below 0.70 was achieved (Aw = 0.534 ± 0.066, n = 3), the top-round beef was vacuum-sealed in mylar-lined pouches and stored at 4 ± 1 °C for one week. Water activity was measured in triplicate after blending all ingredients to ensure shelf stability for the “21% HFLC protein bar” batch (abbreviated as 21% bar), “24% bar” batch, “27% bar” batch, and “30% bar” batch (Aw ± SD = 0.612 ± 0.059, 0.553 ± 0.059, 0.543 ± 0.082, 0.567 ± 0.063, respectively; n = 3) (Appendix A, Table A1).

Beef tallow was heated and cooled under two different temperature conditions, and the samples were set at two different temperatures during sample prototyping to aid in decision-making on ideal setting conditions for creating a firm pemmican bar. One fraction of beef tallow was heated quickly to 85 °C using sealed mason jars submerged in a water bath and held for 3 h, then lowered and held at 65.6 °C overnight before being set in 60 mL plastic sample cups, which were divided into two cooling-phase treatment groups: one set at 3 ± 1 °C and one set at 8 ± 1 °C [25]. After cooling samples overnight in their respective temperature treatment groups at 55% relative humidity, all 60 mL tallow samples were rested at room temperature for 10 min before using a TA-XT2 Texture Analyzer (model: PLUS-UPGRADE, Stable Micro Systems, Godalming, UK) fitted with a 45° conical probe, with the test method employed modified from the standard “Measurement of Firmness of Margarine” sample test project, the test mode being “Compression,” the pre-test probe speed set to 2.00 mm/s, the test speed to 1.00 mm/s, and the post-test speed to 10.00 mm/s. The force applied was set to 100 g with a hold time of 30.00 s, and the collected data included penetration distance (mm), maximum force at the point of penetration (g), and firmness as a function of distance traveled during probe penetration and extraction. The peak force (g) and firmness (g/mm) were measured in triplicate for each temperature group, with firmness defined as the force (g) divided by the distance (mm) traveled by the probe upon penetration. Based on insignificant differences in mean firmness between the two temperature groups (20.55 ± 0.91 g/mm for 3 °C, 21.19 ± 1.07 g/mm for 8 °C; *p* = 0.47), the colder of the two treatments was arbitrarily chosen for the final sample setting (Appendix A, Table A2).

Ingredient preparation involved dicing the dehydrated mango using a Stephan vertical cutter/mixer (model: 19.94, Stephan Machinery Corporation, Columbus, OH, USA) and desiccating the dehydrated top-round beef using a 1.89 L stainless steel Waring blender (model: 503346, Conair LLC, Stamford, CT, USA). The desiccated beef was sieved using a No. 10 2.00 mm sieve (VWR Scientific, West Chester, PA, USA), then combined in a steel mixing bowl with the mango, spices, salt, and melted beef tallow—heated following the same heating cycle used during prototyping—until thoroughly blended with silicon spatulas. The four samples were prepared in batches by rolling to a thickness of 0.635 cm to match the competition product sample thickness, then scored into sectioned bars (5.08 cm × 10.16 cm), which were set at 3 °C overnight. After the sample bars had cooled and set, each bar was vacuum-sealed and stored at 3 °C for 6 days before the week of consumer testing.

### 2.3. Panelist Recruitment

Consumer panelists aged 18–45 years were recruited from the University of Georgia campus and the local community in Athens, Georgia (USA) through an online Qualtrics survey (Qualtrics, LLC, Provo, UT, USA). Panelists were screened for relevant food allergies, low-fat dietary restrictions, and age. Panelists were not screened for prior product familiarity, usage, or frequency. Before each panelist’s voluntary participation in the test, their food allergy status was reconfirmed, and no-sign consent documents were provided along with an explanation of the test. Panelists comprised local walk-ins on campus, primarily faculty, students, and staff. The study was approved by the University of Georgia’s Institutional Review Board (Athens, GA, USA) (IRB Project ID: PROJECT00011621; Review Category: Exempt 6A; approved on 14 March 2025).

### 2.4. Testing Procedure

Samples were prepared by cutting each bar into 3.81 cm squares on the day of the test, dividing them into 60 mL plastic cups with random 3-digit codes, and tempering at room temperature (23 ± 1 °C) for at least 10 min before serving.

Evaluations were conducted under natural white light in six computerized partitioned booths, with each participant tasting all samples over a 1 h testing period. Panelists were given each blind-coded sample monadically in a random order, along with a cup of deionized water for palate cleansing and an expectorant cup. Evaluation ballots were administered via computer monitors within each booth. Fourteen acceptability questions were asked for each sample, followed by a series of demographic questions related to panelist attitudes about consumption habits and ingredient factor preferences regarding the product category (PC) in question, which is described, in the consumer questionnaire, as “snack bars, protein bars, and meal replacement bars.” Panelists were asked to evaluate each sample first by assessing the overall appearance, then the overall flavor (aroma and taste) on a 9-point hedonic scale (1 = dislike extremely; 5 = neither like nor dislike; 9 = like extremely). Saltiness, mango flavor, sweetness, spiciness, and chewiness were evaluated on a 5-point just-about-right (JAR) scale, followed by three intensity questions on a 10-point scale ranging from 0 (none) to 9 (very high) for crumbliness, mouth coating, and aftertaste. Overall liking of the sample and overall texture liking questions were asked at the end of the acceptability questionnaire section on a 9-point hedonic scale, followed by a 9-point Likert scale question on the likelihood of consuming the sample in a whole serving and purchase intent, ranging from 1 (extremely unlikely) to 9 (extremely likely). Demographic information (age group and gender) was collected after all samples were evaluated, followed by check-all-that-apply (CATA) questions on the frequency of PC consumption, the typical occasion for PC consumption, and the primary reasons for PC consumption. The last demographic question included a 5-point ranking of 11 ingredient factors related to label claims, ranging from 1 (completely undesirable) to 5 (very desirable). Ingredient factors assessed included “high protein,” “no added sugar,” “low in polyunsaturated fats,” “organic ingredients,” “all-natural ingredients,” “minimal ingredient list,” “high satiety (keeps you full for hours),” “dairy-free,” “grain-free (free of soy, corn barley, millet, rice, etc.),” “hypoallergenic (free of soy, milk, wheat, shellfish, fish, tree nuts, peanuts, sesame, egg), and “hyper-digestible.” Consumer evaluation, sensory questionnaire administration, and data collection were performed using Compusense Cloud (Version 25.0.32593; Compusense, Inc., Guelph, ON, Canada).

### 2.5. Data Analyses

Collected data were analyzed using XLSTAT (Version 2025.2.0; Addinsoft, New York, NY, USA). Overall differences in consumer acceptability, assessed through hedonic attribute ratings and intensity scores of sensory attributes, were analyzed using one-way ANOVA, with significant differences (*p* ≤ 0.05) identified and further analyzed using Fisher’s Least Significant Difference (LSD) post hoc test when appropriate. This analysis, including multiple pairwise comparisons, was performed using the sample names as the independent factor and hedonic attribute and intensity scores as the dependent variables. Two-way ANOVA and pairwise comparisons were performed for ingredient factor rankings within gender and age groups separately, with age groups combined into two (18–24 years, 25–45 years old) due to insufficient sample size in the third age group (35–45 years old; n = 3). A series of 2-way ANOVA analyses, with the same post hoc analysis, was performed on overall sample liking (dependent variable), with gender (n = 101; 1 participant omitted gender information) and age group (n = 102) as independent factors. A Kruskal–Wallis test was performed on purchase intent and full-serving likelihood questions, treating sample names as independent variables. Multiple pairwise comparisons were performed using Dunn’s test at 5% level of significance (*p* ≤ 0.05). JAR questions related to sample attributes were analyzed using a penalty analysis to identify positive and negative drivers of each sample’s overall liking. Demographic check-all-that-apply (CATA) and multiple-choice questions were analyzed using descriptive statistics to determine overall frequencies and gender- and age-specific frequencies.

Internal preference mapping (IPM) was performed using each participant’s overall liking as an active variable. IPM is a principal component analysis (PCA) where the vector loadings correspond to consumers’ overall liking scores. A correlation matrix was used during the analysis. Supplementary variables included overall texture, overall appearance, overall flavor, aftertaste, mouth coating, crumbliness, likelihood of a whole serving, and purchase intent. Observations c17, c19, c32, and c81 were excluded from this analysis because they had identical overall liking scores across all five samples. A second PCA was conducted using the “clean label” data. All nine attributes were treated as objects in the PCA matrix, with the participants’ data for these attributes serving as observations. Female participants were designated as “F” in the data matrix, while male participants were labeled as “M”. A correlation matrix was used during the analysis. Gender (male or female) and age (<25 y or >25 y) were included as supplementary variables.

## 3. Results and Discussion

### 3.1. Consumer Demographic Characteristics

Table 2 summarizes the demographic characteristics of the study participants (n = 102) and the frequency of CATA responses across PC consumption occasions. The participants included 45 females (44%), 56 males (55%), and 1 participant (<1%) who did not respond. The age groups represented were 66 participants aged 18–24 (65%) and 36 participants aged 25–45 (35%). The most common frequency pattern observed for PC consumption was “a few times a month” (42% of participants), followed by “few times a week” (25% of participants). The most frequently reported occasions for PC consumption were “afternoon snack” (47% of participants), followed by “breakfast” (37%) and “post-workout recovery” (33%). The most frequently checked reason for product category consumption was “convenience on the go” (78% of participants). The second-most frequently checked reason was “hunger management/snacking” (60%), followed by “taste/flavor enjoyment” (37%). These findings are similar to those from a study on health-conscious Brazilian snack bar consumers, in which the most critical factors in purchasing snack bars were price, flavor, and package attributes such as brand name, graphic design, and colors [26]. Likewise, a study of New Zealand consumers found that price, taste, convenience, and brand were the four most frequently cited attributes when making snack food purchase decisions [27].

### 3.2. Consumer Acceptability

Mean hedonic responses to each sample and mean intensity ratings for all samples are summarized in Table 3. None of the samples were liked, suggesting that the study participants were unfamiliar with or infrequent buyers of HFLC bars. Overall appearance was not significantly different among the evaluated samples (*p* > 0.05), but across the four prepared samples, it increased linearly with increasing lean-beef content and decreasing fat content. Overall flavor and aroma liking were rated significantly higher for all prepared samples compared with the commercial bar (*p* ≤ 0.05). Still, no significant difference in overall flavor was observed among the four prepared samples (*p* > 0.05). The 30% bar was rated highest for flavor, while the 21% bar was rated lowest, with a consistent downward trend in average liking as the fat content of each bar increased. While the commercial bar ranked lowest in overall liking, one must account for differences in flavor presentation when evaluating overall flavor liking. A generalized statement about preferences for sweet–spicy flavors over sweet–savory flavors could be made based on this observation in the context of HFLC protein bars. Overall texture was not significantly different among the evaluated samples (*p* > 0.05). The commercial bar had the lowest average liking for texture, while the 30% bar had the highest. The intensity of crumbliness was not significantly different between the 30% bar, the 24% bar, and the 21% bar (*p* > 0.05), but the mean intensities for each bar within this group were significantly lower (*p* ≤ 0.05) when compared to the 27% bar and the commercial bar. The highest mean intensity of crumbliness was observed for the 27% bar, which was not significantly different from the intensity of crumbliness observed in the commercial bar (*p* > 0.05). The intensity of mouth coating was not significantly different among the samples (*p* > 0.05). Aftertaste intensity was not significantly different among the four prepared samples (*p* > 0.05). It could not be compared with the commercial bar because of differences in the peppers used in the formulation, which contributed to a spicier aftertaste.

Overall liking was significantly lower for the commercial bar than for all prepared samples (*p* < 0.001), with an average score corresponding to “Dislike Moderately.” All prepared samples had comparable overall liking scores (*p* > 0.05), corresponding to “Dislike Slightly.” The likelihood of consuming a whole serving of product was significantly lower for the commercial bar than for all prepared samples (*p* ≤ 0.01). In contrast, the prepared samples did not differ considerably from one another (*p* > 0.05). This same pattern was observed for the five-point rating of purchase likelihood, where the commercial bar scored significantly lowest, close to the “Extremely Unlikely” anchor (*p* ≤ 0.01), and all prepared samples were not significantly different from one another (*p* > 0.05), except for the 21% bar, which did not differ significantly from the commercial bar (*p* > 0.05). For the categories overall liking, full-serving likelihood, and purchase intent, mean scores increased, corresponding with higher protein content in the prepared sample group. The commercial bar’s different flavor profile and processing method were likely the main drivers of its lower acceptability, as evidenced by differences in texture, crumbliness, and overall flavor liking compared to the prepared samples.

Table 4 summarizes the results by age group and gender. No significant differences were observed (*p* > 0.05) between males and females for each sample’s overall liking. No significant difference in overall liking was observed between the two age groups for each sample (*p* > 0.05) except for the 24% and 30% bars, which were liked significantly more by the older age group above 25 years old (*p* ≤ 0.05), with overall liking for all four prepared samples trending higher in this group. This could indicate a greater implicit preference for or familiarity with high-fat or meat products among older adults than among adults under age 25. Differences in sample liking across age groups may arise from increased preference for fatness-mediated sensory attributes, such as thickness and creaminess, in older adults [28]. However, a 2020 survey of 1000 American adults found mixed and changing opinions on dietary fats, with people under 45 more likely to search for full-fat products than those aged 45–64, and more likely to say full-fat products were healthier than low-fat versions [29]. The limited age range in this study hinders comparability with other studies that use older participant populations. Still, previous research has identified differences in older and younger generations’ preferences for protein bars [28,30]. The overall low acceptability of all samples, including the commercially available bar, indicates a lack of familiarity with the product type. This is supported by the lack of commercially available “pemmican”-style protein bars on the market, as well as low familiarity with meat bar snacks—defined as snacks made of dried meat, fruit, and nuts—among U.S. consumers [19].

### 3.3. JAR Attributes

**Figure 1:**
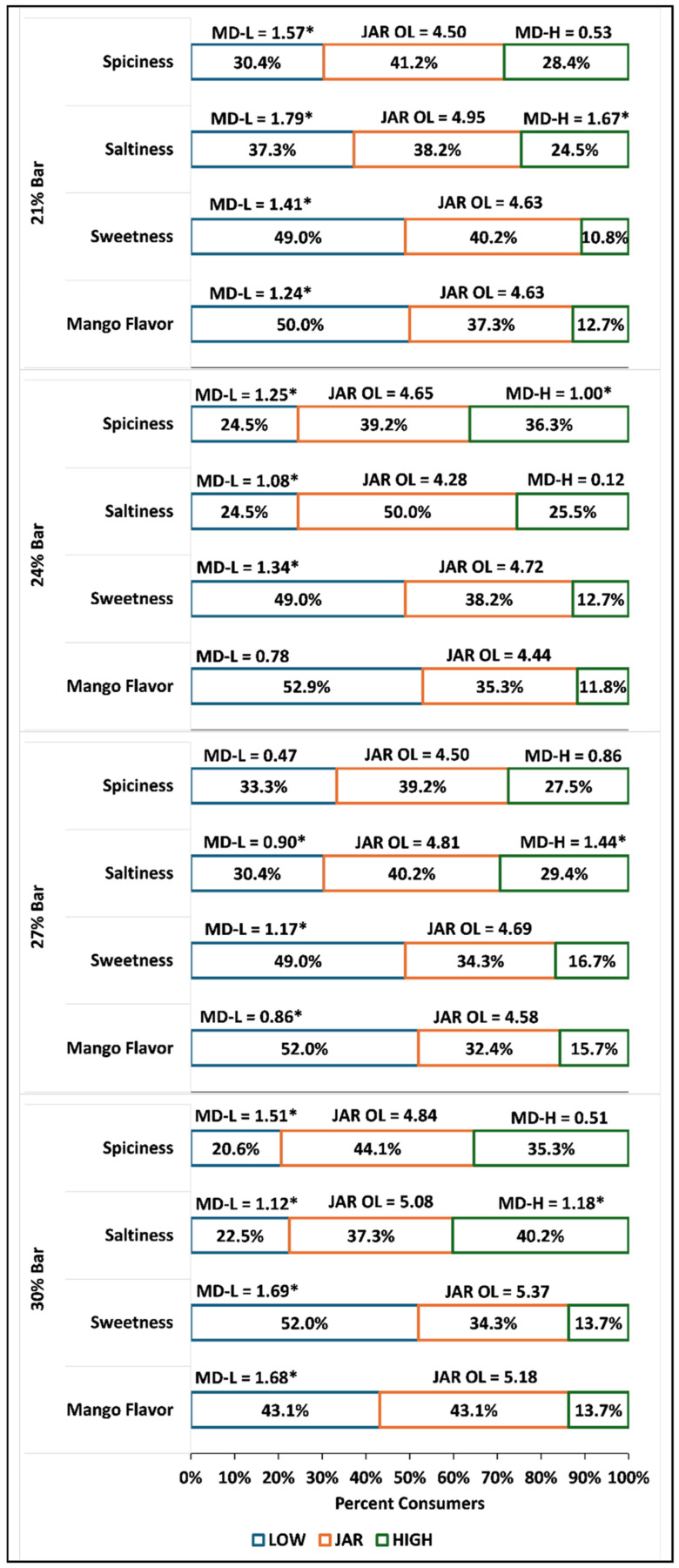
Results for the four JAR flavor attributes for the four experimental samples. The counts within each bar show the percentage of participants who rated that attribute as low (blue), just about right (orange), and high (green). MD-L = Mean Drop Low; MD-H = Mean Drop High; JAR-OL = JAR category overall liking score. * Significant at *p* ≤ 0.05.

Figure 1 shows the results of the JAR penalty analysis. Data for the commercial bar was excluded from this analysis. Overall sweetness and mango flavor were too weak to elicit more than a 50% JAR response and caused a significant average decrease for the 21% bar (*p* ≤ 0.05 for mango flavor; *p* ≤ 0.001 for sweetness), 27% bar (*p* ≤ 0.05 for mango flavor; *p* ≤ 0.05 for sweetness), and 30% bar (*p* ≤ 0.001 for mango flavor; *p* ≤ 0.05 for sweetness).

For the most-liked sample, the 30% bar, the most significant mean differences were observed for “weak”-rated sweetness, mango flavor, and spiciness (*p* ≤ 0.05). Spiciness that was rated as “strong” did not significantly impact mean liking, despite being rated as “strong” by 36 participants for this sample (*p* > 0.05). For all prepared samples, spiciness was evaluated similarly across all fat content levels, indicating that spiciness can be a flavoring agent optimized independently of fat concentration. Saltiness significantly reduced mean liking when rated as weak or strong (*p* ≤ 0.05), but a larger proportion of participants rated it as strong, indicating that it could be reduced in the 30% bar sample.

Other flavor combinations can significantly affect saltiness in a food matrix. In a study on saltiness perception of soup with varying levels of fat and constant piperine concentration (the compound responsible for pepperiness in black pepper), Moss et al. [31] found that piperine increased perceived saltiness, bitterness, and spiciness, while additional fat content reduced bitterness and spiciness but increased sweetness, aftertaste intensity, and overall liking. Interestingly, Moss et al. [31] found no effect of extra fat content on perceived saltiness. These results contrast with the JAR and overall liking results of this study, in which saltiness was the least intense in the two highest-fat samples. In contrast, the most-liked and least-liked samples had the highest fraction of participants who rated saltiness as “strong,” and this fraction decreased linearly with increasing fat content. This evidence, when taken together with other studies’ findings (both decreased and increased salt intensity with fat addition) [32,33], indicates that more research on fat content and salt perception is needed, with scrutiny of the fat type used and the food matrix context. Spiciness did not noticeably reduce with increasing fat content in this study. A study by Carden et al. [34] on cheese sauces with varying fat contents found that low concentrations of capsaicin—the compound responsible for “spicy” flavor sensation in capsicum peppers—did not differ in perception across different fat levels. Still, it exhibited greater heat intensity in low-fat sauces relative to full-fat sauces at 1.2 ppm capsaicin. This may indicate that the habanero concentrations used in the formulations for the current study were low enough not to be affected by the changing fat levels. The 24% bar had the highest proportion of salt acceptability (51 JAR responses), suggesting an optimal range for fat–salt synergy. Moss et al.’s findings on sweetness and fat synergy were only marginally confirmed by the increasing JAR response frequency for sweetness with increasing fat content observed in this study, with the results not being statistically significant (*p* > 0.05) [31]. Sweetness and fat, as well as salt and fat, synergies should be further explored across a broader range of fat, sugar, and salt concentrations, with consumers’ sweet/salty preferences taken into account. Bitter-taste-sensitive individuals, for example, prefer higher concentrations of fat and sugar together (30% fat to 15% sucrose *w*/*w*) compared to non-bitter-sensitive individuals (3.3% fat to 10% sucrose *w*/*w*) for optimal liking [35]. Given these findings, utilizing high-fat food matrices to reduce overall salt concentration may be viable only within a given range of fat concentration, where a particular volume may enhance saltiness acceptability but mask it in elevated concentrations.

The interaction between fat and sweetness in this study needs to be further explored using a broader range of sugar concentrations, given the overall weakness in perceived sweetness across all samples. Drewnowski and Schwartz [36] found, in a comparison of perceived sweetness and fat content in custard, that increasing sucrose levels dramatically decreased perception of fat, independent of fat stimulus content, with the lowest fat content being perceived in the highest sucrose solution. Furthermore, increased fat content did not significantly affect fat perception, whereas sucrose concentration did via suppression of oral fat perception. This may be due to both changes in texture due to sugar’s crystalline nature and the sweetness stimulus masking oral fat “mouthfeel.” Sweetness intensity increased with higher sucrose content and only marginally with increased fat content (15% compared to 25% and 35% *w*/*w*), according to Drewnowski and Schwartz’s findings; however, a lack of overall acceptability data remains, preventing the clear establishment of a relationship between varying fat content and increasing sugar content beyond “optimal” sweetness.preventing the establishment of A separate study comparing the impacts of sugar and fat perception in solid versus liquid foods found that a higher fat content was preferred in solid food matrices (35% *w*/*w*) compared to liquid (20% *w*/*w*) while the optimal sugar level remained approximately equal (around 16%) [37]. Interestingly, this same study found that fat perception in solid foods was impaired compared to liquid foods, so that higher fat content in solid food systems was preferred but not accurately perceived as increased by the consumer panel [37]. While fat can be explored to increase perceived sweetness and overall acceptability, other interacting sensory components can also be leveraged. Odor-induced sweetness enhancement is a novel strategy for enhancing perceived sweetness without increasing sugar content. In a study by Ge et al. [38] on mango flavor and aroma at different sucrose concentrations in a beverage context, sugar could be reduced moderately while maintaining sweetness using mango aroma. The maximum overall liking found was at 4.28% sucrose and 0.57% mango flavor concentration, with optimal odor-induced sweetness enhancement observed in medium-to-low sucrose concentration solutions (0.25% mango flavor with 2.24% sucrose content) [38].

### 3.4. Internal Preference Mapping (IPM) of Consumer Attributes

**Figure 2:**
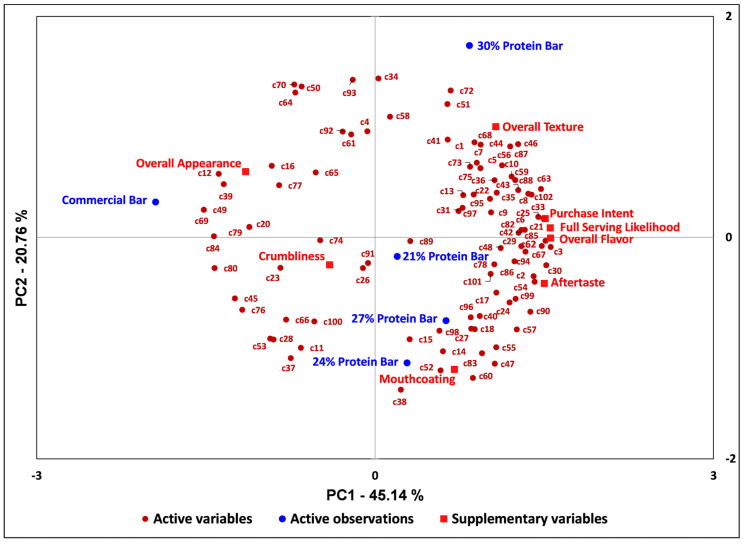
Internal preference map (IPM) of participants and sample liking. All points labeled “c#” (active variables) represent a consumer’s overall liking of the samples (active observations). The supplementary variables included overall texture, overall appearance, overall flavor, aftertaste, mouth coating, crumbliness, likelihood of a whole serving, and purchase intent.

Figure 2 shows the internal preference map (IPM) for consumer attribute scores across samples. The first principal component (PC1) explains 45.14% of the variance in the sample acceptability results, while the second principal component (PC2) explains 20.76%. The preference map indicates that most consumers favored either the 21%, 24%, or 30% bars. The commercial bar was the most dissimilar in liking and associated attributes, with a smaller segment of consumers clustering around this sample. The larger cluster to the right of the preference biplot suggests general liking of the prepared samples and a general disliking of the commercially available sample, with appearance and crumbliness being attributes more associated with the commercial bar and potential key factors for liking in this smallest consumer segment. The 30% bar scored highest for mean overall liking, flavor, and texture, but is more closely associated with overall texture on the biplot. This indicates that consumers clustering closer to the 30% bar compared to the other prepared bars are likely influenced more by texture acceptance in their overall liking, and that texture was the main attribute that distinguished the overall perception of the 30% bar from the other samples.and that texture was the primary attribute that differentiated Crumbliness is in the opposite direction of overall texture, indicating a negative potential relationship between the two attributes. Higher crumbliness in the bars decreased texture acceptability.

Innova [39] reports that texture claims are attracting younger consumers, with snack bars ranking as the third-fastest-growing category in this space. New snack bar product launches are prioritizing texture, with “chewy” as the traditional protein bar profile and “crispy,” “crunchy,” and “soft” rising in popularity [4]. Improving the texture acceptability of meat snack bars is imperative for growing young consumer bases in this product category. Given the contrasting JAR results for the most-liked and least-liked samples in this study as well as the results of the internal preference map, focusing on texture optimization and consumer segmentation based on texture preferences would be viable strategies for improving consumer acceptability of HFLC bars, particularly as it is impacted by fat content and its synergy with other ingredients in a solid food product. Dietary fat acceptability appears unique to the given food system, based on its impacts on texture, mouthfeel, and olfaction, with primary detection methods such as mouthfeel and texture making it challenging to optimize perception of fat alone across different food systems [37]. For the product category in question, incorporation of “clean-label”-compliant starches, such as whole wheat flour, can markedly improve the overall acceptability of meat bars by maintaining softness and enhancing crispiness by creating more pits and holes in the meat bar matrix [21]. Methods of incorporation, tempering, and setting of beef tallow in an HFLC bar should also be explored to improve bar firmness and portability, given the significant impact of crumbliness on overall liking of the commercially available HFLC bar.

### 3.5. “Clean Label” Ingredient Factor Preferences

**Figure 3:**
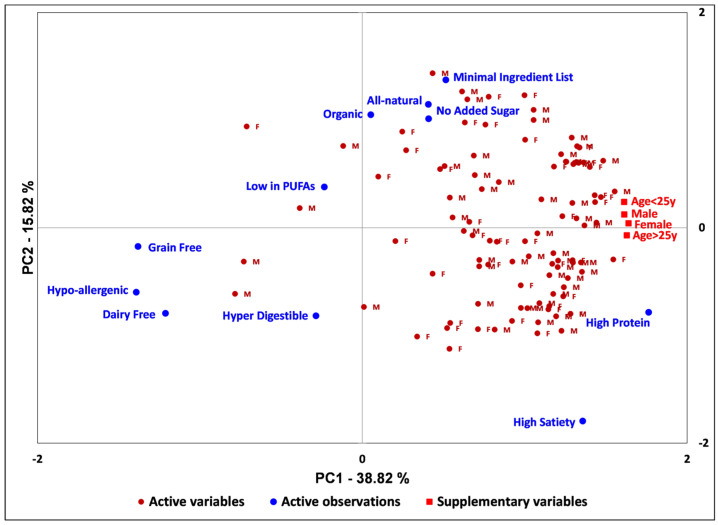
PCA biplot of the “clean label” ingredient factor attributes measured on a 5-point Likert scale. Active variables = consumer scores for the “clean label” ingredient factor attributes, Active observations = “clean label” ingredient factor attributes, Supplementary variables = gender and age groups.

Figure 3 shows a PCA biplot that illustrates their importance in making purchase decisions for the product category (PC). The label claim with the highest average desirability was “high protein,” with a mean of 4.40, followed by “high satiety” at 3.95, “minimal ingredient list” at 3.77, and “all-natural” at 3.75. Compared to all other claims, “high protein” was the only label claim with a minimum ranking response of 3. In contrast, all other claims had a minimum reaction of 1, indicating that no panelists in the assessed population found high protein content in the PC to be undesirable. These findings confirm modern consumer choice trends, where the “health halo” of protein has driven up to 56% of U.S. consumers to actively seek higher protein intake, with a third using fortified foods, particularly “permissible indulgences” such as dessert-flavored protein bars [4]. Similar results were found by Rovai et al. [40] in an online survey of U.S. consumers of protein-fortified foods (n = 405), in which consumers asked about ingredient preferences placed the highest importance on protein amount, followed by protein type. Furthermore, consumers were skeptical of unrecognizable ingredients such as gums and stabilizers in protein beverages, desiring fewer additional ingredients overall beyond recognizable protein and natural sweeteners. Aligning both with the importance placed on “high satiety” and the high frequency of “hunger management” as a reason for PC consumption found in this study, Pinto et al. [26] found that consumers are willing to pay more for snack bars that offer higher satiety, citing dissatisfaction with conventional 20 g protein bars on the market. The following top-four ingredient factor rankings confirm previous findings on consumer perceptions of clean labels for generalized food products.

Cao and Miao [10] found in an online consumer survey (n = 346) that the most essential “clean label” attribute by mean score was “less/minimally processed,” followed—in order of importance—by “ingredients that you recognize,” “ingredients’ functions that you know,” “absence of artificial additives,” “made with simple ingredients,” and “shorter list of ingredients.” Regardless of the scientific definition, surveyed consumers associated “clean label” attributes of elimination of undesired ingredients, less processing, and utilization of recognizable, familiar ingredients with healthiness benefits, social responsibility, and higher sensory appeal. The near-equal and higher importance placed on “minimal ingredient list” and “all-natural” ingredients compared to “organic” in this study’s results highlights Cao and Miao’s findings on the discrepancies between industry and consumer definitions of “clean label,” where “organic,“ “natural,” and “free from” were perceived by consumers as subcategories of “less/minimally processed” labels, revealing that “clean label” seekers are looking for omission of undesirable ingredients and reduced processing before they are won over by supplemental claims such as “organic” [10].

The fifth-highest-ranked label claim was “no added sugar,” with a mean of 3.65. This supports findings that sugar reduction has become a global phenomenon, with three in five U.S. consumers working to reduce their overall intake due to concerns about blood sugar spikes, inflammation, and weight gain [41]. Childs et al. [42] also found that consumers of protein-fortified meal replacement products seek out “low-sugar” and “natural” claims on packaging. The lowest-valued label claim was “grain-free,” with a mean value of 2.73. Comparable mean values close to this include 2.79 for both “dairy-free” and “hypoallergenic.” These label claims, on average, fall somewhere between undesirable and no preference. The low ranking for “grain-free” could indicate that this is not a concern for the sample population, or that it is even a deterrent in the absence of grains. There were no significant effects of gender and age or their interaction (gender by age) except for “dairy-free” in gender and “organic” in age (Table S1). The insignificance of gender and age is also evident in the PCA biplot (Figure 3), where the gender and age variables are clustered together on the right side. Although this study’s age group pool has limitations, these results can be compared with findings from U.S. consumer surveys on protein-fortified food choices. Keefer et al. [43] found that among three consumer segments, the group with the highest average age (37.5 years) also had the highest proportion of low-sugar and low-carb dieters. These findings may correspond to the slightly higher rankings observed for “low in PUFAs” and “grain-free” in the oldest age group in this study. In a UK study specifically analyzing consumer decision-making for meal replacement products, perceptions of ultra-processing were a key purchase driver, second only to price, in meal replacement bars, with 83% of participants (n = 302) being familiar with the term “ultra-processed foods” [44]. Aligning with the results found in this study, Rouse et al. found consumers (mean age of 38.8 years) were most likely to select meal replacement products that were minimally processed, met daily requirements for vitamins and minerals, and contained claims of “high protein,” while claims of “low fat” or “reduced calorie” resulted in lower likelihood of product selection [44]. Interestingly, recent events and the findings of this study indicate a growing consensus towards defining clean label among consumers, industry players, and government entities. This is evidenced by recent changes to the U.S. dietary guidelines, which emphasize a diet prioritizing “real” versus processed foods, defined as whole or minimally processed and containing minimal ingredients while omitting added sugars, industrial oils, and artificial additives [45]. While younger generations of consumers may be more familiar with low-fat food products and formulations, changing sentiment on the nutritional benefits of whole-fat products, natural protein, and minimally refined carbohydrates highlights the need for food scientists to optimize sensory performance of traditionally prepared foods showcasing natural animal and plant fats, with this study providing some of the groundwork specific to beef tallow—a natural animal fat mentioned by the new dietary guidelines for Americans as an option for increasing whole-food healthy fat sources [46]. The conjoint emphasis of the new dietary guidelines on adequate protein at every meal, as well as minimal ingredient processing [45], will likely be a challenge for traditional snack products, a realization made in carrying out this study, where time-consuming and labor-intensive hand processing was required for developing a dehydrated, non-ultra-processed beef protein base. Further investigation into efficient methods for processing snack food ingredients is required to meet both consumer demands and changing nutritional recommendations for minimally processed foods that do not sacrifice convenience, shelf stability, ease of manufacturing, or palatability.

## 4. Limitations and Conclusions

### 4.1. Limitations

Limitations to this study include the representativeness of the general population within the sample, the preparation of the food samples evaluated, and the attributes assessed. Participants were recruited exclusively from the University of Georgia campus, meaning the majority were local to the area, had a higher-education background, or had a professional background with high levels of education. Furthermore, participants aged 45 years or older were not recruited to maintain the specificity of the findings to the preferences of the younger adult age group. The product category’s limited prevalence in mainstream food markets limited the data available for extrapolating product familiarity and its potential impact on product liking. For this reason, only a limited number of commercial products were available for comparison. The commercial product presents its own limitations due to differences in flavor profile from the prepared samples, which prevent specific conclusions about how ingredient processing, final texture, and fat content impact acceptability independent of overall flavor. Low familiarity among the untrained consumer panel with attribute descriptors related to fat content, such as “mouthfeel,” may have led to variability in responses and in the correlation between fat content and overall liking. Another limitation in correlating fat content to overall liking stems from the lack of clear data obtained on how beef tallow processing methods determine the final product texture, independent of total fat content.

### 4.2. Conclusions

This study effectively explored general consumer and preferences towards high-fat, low-carbohydrate protein bars, using an under-investigated “pemmican”-inspired formulation to guide future sensory optimization and consumer-centric product development of snack bars, meal replacement bars, and protein bars, epitomizing consumer-defined “clean label” ideals of minimal, natural ingredients with minimal processing. Higher-fat-containing HFLC bars are less acceptable than those with higher lean-beef content, with lack of product familiarity/usage, texture, and flavor suppression as key drivers of lower acceptability. Synergistic flavors, such as sweetness and spiciness, can be used to improve the acceptability of high-fat products, which rely on higher fat content for shelf stability, cohesiveness, and whole-food-based nutrition. Gaps in the previous literature on fat perception in solid foods and the texture impacts of fat content identified in this study highlight the need for further research on methods for incorporating minimally processed animal fats into shelf-stable snack food products. Methods of animal fat processing play a key, yet under-investigated, role in fat’s contribution to overall product functionality—especially texture—and acceptability, beyond its impact on total fat content. Likewise, further exploration is needed into the impacts of novel processing methods on the texture and shelf life of non-ultra-processed protein sources. The importance of ingredient factors for snack bar, meal replacement bar, and protein bar consumption behavior aligns with current market trends and past research, which highlight a general focus on protein content, satiety, and minimal, all-natural ingredients. Less-prioritized label claims, such as “grain-free,” “dairy-free,” and “hypoallergenic,” may appeal more to niche consumer segments rather than the general young adult population. Food product developers should prioritize exploring traditional methods of food preservation, preparation, and natural ingredient functionality showcased in pemmican bar formulations to better inform modern food processing methods in meeting consumer demands for convenience foods that provide adequate sensory appeal without compromising on ideals of high protein content, shorter ingredient lists overall, minimal processing, and whole-food-based ingredients.

## Figures and Tables

**Table 1:** HFLC protein bar formulations for a single batch.

Ingredient	Manufacturer	21% Protein HFLC Bar g (% *w*/*w*)	24% Protein HFLC Bar g (% *w*/*w*)	27% Protein HFLC Bar g (% *w*/*w*)	30% Protein HFLC Bar g (% *w*/*w*)
Dried Beef	Publix Super Market, Inc., Lakeland, FL, USA	632.43 (30.69)	690.45 (33.51)	742.93 (36.05)	790.95 (38.38)
Grass-fed Beef Tallow	The Fat Lady Tallow, Palmyra WI, USA	1058.27 (51.35)	971.24 (47.13)	892.51 (43.31)	820.48 (39.82)
Dried Mango	Anna and Sarah, Somerset, NJ, USA	316.22 (15.35)	345.23 (16.75)	371.47 (18.03)	395.48 (19.19)
Black Pepper	Badia Spices, LLC., Doral, FL, USA	13.45 (0.65)	13.45 (0.65)	13.45 (0.65)	13.45 (0.65)
White Pepper	Felicific, Inc., Queens, NY, USA	13.45 (0.65)	13.45 (0.65)	13.45 (0.65)	13.45 (0.65)
Garlic Powder	Badia Spices, LLC., Doral, FL, USA	13.45 (0.65)	13.45 (0.65)	13.45 (0.65)	13.45 (0.65)
Habanero Powder	Sonoran Spice, Scottsdale, AZ, USA	13.45 (0.65)	13.45 (0.65)	13.45 (0.65)	13.45 (0.65)
Sea Salt	SoSalt SPA, Trapani, Italy	7.00 (0.34)	7.00 (0.34)	7.00 (0.34)	7.00 (0.34)
Total		2060.70 (100)	2060.70 (100)	2060.70 (100)	2060.70 (100)

**Table 2:** Demographic and consumption data of the participants related to the product category (PC) snack bars, protein bars, and meal replacement bars (n = 102).

Description	Total	Total (%)
Participants	102	100
Gender		
Female	45	44
Male	56	55
Did not answer	1	<1
Age in years		
18 to <25	66	65
>25	36	35
Consumption pattern		
Frequency		
Daily	3	3
Few times a week	26	25
Once a week	16	16
A few times a month	43	42
Rarely	18	18
When consumed		
Breakfast	38	37
Mid-morning snack	23	23
Lunch replacement	10	10
Afternoon snack	48	47
Pre-workout fuel	15	15
Post-workout recovery	34	33
Dinner replacement	1	1
Late-night snack	23	23
Anytime	21	21
Reason for consumption		
Convenience/On the go	80	78
Weight management	16	16
Meal replacement	17	17
Post-workout recovery	27	26
Hunger management	61	60
Nutritional supplementation	34	33
Flavor enjoyment	38	37
Other—no description	2	2

**Table 3:** Mean scores ± standard deviation for “hedonic,” “diagnostic,” and “other product preference” responses for the samples.

Attribute	Test Statistic	21% Protein HFLC Bar	24% Protein HFLC Bar	27% Protein HFLC Bar	30% Protein HFLC Bar	Commercial Bar
Hedonic Responses (9-point Hedonic Scale)	ANOVA F-value (4, 505)					
Overall Appearance	1.38	3.86 ± 1.71	4.03 ± 1.76	4.06 ± 1.67	4.16 ± 1.77	4.43 ± 2.08
Overall Flavor	8.44 *	4.16 ^a^ ± 1.80	4.31 ^a^ ± 2.03	4.38 ^a^ ± 1.89	4.54 ^a^ ± 2.12	3.15 ^b^ ± 1.77
Overall Texture	0.60	3.78 ± 1.74	3.82 ± 1.80	3.76 ± 1.71	4.06 ± 1.90	3.68 ± 2.13
Overall Liking	7.93 *	3.87 ^a^ ± 1.89	3.98 ^a^ ± 2.02	4.11 ^a^ ± 1.90	4.35 ^a^ ± 2.04	2.92 ^b^ ± 1.96
Diagnostic Responses (0–9 Numerical Scale)						
Crumbliness	5.25 *	3.45 ^c^ ± 1.94	3.73 ^c^ ± 2.11	4.67 ^a^ ± 2.18	3.79 ^bc^ ± 2.02	4.32 ^ab^ ± 2.53
Mouth Coating	0.50	4.30 ± 2.43	4.22 ± 2.35	4.42 ± 2.34	3.94 ± 2.13	3.95 ± 2.48
Aftertaste	6.67 *	5.15 ± 2.16	5.39 ± 2.31	5.40 ± 2.07	5.23 ± 2.26	3.99 ± 2.72
Other Preference Responses (5-point Likert Scale)	Kruskal–Wallis K-value					
Full-Serving Likelihood	23.63 *	2.76 ^a^ ± 1.82	2.83 ^a^ ± 1.82	2.88 ^a^ ± 1.74	3.03 ^a^ ± 1.86	2.12 ^b^ ± 1.65
Purchase Intent	21.36 *	2.23 ^ab^ ± 1.48	2.41 ^a^ ± 1.63	2.47 ^a^ ± 1.60	2.63 ^a^ ± 1.68	1.78 ^b^ ± 1.35

* Significant F-value or K-value (*p* ≤ 0.05). ^abc^ Common letter (superscript) in each row for samples indicates no statistical difference (*p* > 0.05).

**Table 4:** Mean scores for “overall liking” and “purchase intent” responses separated by gender and age.

Description	Two-Way ANOVA	21% Protein HFLC Bar	24% Protein HFLC Bar	27% Protein HFLC Bar	30% Protein HFLC Bar	Commercial Bar
Gender (n = 101)	F-value (1, 504)					
Overall Liking	0.11 ^NS^	*p* = 0.232	*p* = 0.317	*p* = 0.715	*p* = 0.988	*p* = 0.276
Female (n = 45)		4.11 ± 1.98	4.18 ± 2.12	4.00 ± 2.09	4.33 ± 2.15	2.64 ± 1.77
Male (n = 56)		3.64 ± 1.79	3.78 ± 1.93	4.14 ± 1.73	4.34 ± 1.98	3.07 ± 2.04
Age (n = 102)	F-value (1, 500)					
Overall Liking	4.25 *	*p* = 0.486	*p* = 0.078	*p* = 0.518	*p* = 0.053	*p* = 0.659
<25 y (n = 66)		3.77 ± 1.97	3.73 ± 2.00	4.02 ± 1.84	4.08 ± 2.01	2.98 ± 2.02
>25 y (n = 36)		4.06 ± 1.74	4.44 ± 2.01	4.38 ± 2.04	4.86 ± 2.03	2.80 ± 1.86

^NS^ not significant; * significant at *p* ≤ 0.05.

## Data Availability

The raw data supporting the conclusions of this article will be made available by the authors on request.

## Supplementary Materials

The following supporting information can be downloaded at https://www.mdpi.com/article/10.3390/foods15030551/s1. Table S1. “Clean label” attribute scores (measured on a 5-point Likert scale) regarding participants’ preferences for snack bars, meal replacement bars, and protein bars.

## Appendix A

**Table A1:** Water activity of initial top-round beef and final HFLC bars.

Sample ID	Average A_w_ ^1^	SD ^2^
TR Batch ^3^	0.534	0.066
21% Protein Bar	0.612	0.059
24% Protein Bar	0.553	0.059
27% Protein Bar	0.543	0.082
30% Protein Bar	0.567	0.063

^1^ A_w_ = peak force in grams (g). ^2^ SD = standard deviation. ^3^ TR = top-round.

**Table A2:** Firmness tests for beef tallow.

Sample ID	Mean PF ^1^ (g)	PF (g) SD ^2^	Mean Firmness (g/mm)	Firmness (g/mm) SD
T-3C ^3^	0.534	0.066	20.547	0.906 ^NS^
T-8C ^4^	0.612	0.059	21.193	1.072 ^NS^

^1^ PF = peak force in grams (g). ^2^ SD = standard deviation. ^3^ T-3C = tallow set at 3 °C; ^4^ T-8C = tallow set at 8 °C; ^NS^ not significant (*p*-value = 0.469).
