# Supplementary material for: Consumer Insights into “Clean Label” High-Fat, Low-Carbohydrate Protein Bars
Source: Foods. 2026 Feb 4;15(3):551. doi: 10.3390/foods15030551 (PMC12897269; doi:10.3390/foods15030551)
Supplement: Supplementary file 1 [file foods-15-00551-s001.zip › foods-4093201-supplementary.pdf]

Table S1. "Clean label" attribute scores reported as mean ± standard deviation (measured on a 5-point Likert scale) regarding participants' preferences for snack bars, meal replacement bars, and protein bars.

Description	High Protein	No Added Sugar	Low in PUFAs	Organic	All Natural	Minimal Ingredient List	High Satiety	Dairy-free	Grain-free	Hypo-allergenic	Hyper-Digestible
Gender (n = 101)	$p = 0.313$	$p = 0.335$	$p = 0.370$	$p = 0.696$	$p = 0.794$	$p = 0.414$	$p = 0.272$	$p = 0.001$***	$p = 0.242$	$p = 0.109$	$p = 0.640$
Female (n = 45)	4.32 ± 0.70	3.76 ± 0.99	3.44 ± 0.81	3.50 ± 0.99	3.73 ± 0.88	3.81 ± 0.93	4.08 ± 0.88	3.14 ± 0.84	2.84 ± 0.64	2.95 ± 0.72	3.32 ± 0.74
Male (n = 56)	4.47 ± 0.69	3.55 ± 1.11	3.28 ± 0.90	3.43 ± 0.87	3.68 ± 0.86	3.64 ± 0.99	3.85 ± 1.05	2.48 ± 0.95	2.64 ± 0.93	2.63 ± 1.09	3.40 ± 0.87
Age (n = 101)	$p = 0.93$	$p = 0.973$	$p = 0.502$	$p = 0.026$*	$p = 0.051$	$p = 0.058$	$p = 0.953$	$p = 0.681$	$p = 0.870$	$p = 0.386$	$p = 0.305$
< 25 y (n = 65)	4.40 ± 0.73	3.66 ± 1.06	3.30 ± 0.82	3.68 ± 0.85	3.88 ± 0.86	3.91 ± 0.91	3.96 ± 0.97	2.85 ± 0.95	2.73 ± 0.84	2.87 ± 1.06	3.45 ± 0.77
≥ 25 y (n = 36)	4.39 ± 0.64	3.65 ± 1.05	3.42 ± 0.94	3.25 ± 1.00	3.53 ± 0.84	3.53 ± 1.00	3.97 ± 1.03	2.77 ± 0.97	2.75 ± 0.77	2.70 ± 0.71	3.27 ± 0.88
Gender × Age (n = 101)	$p = 0.586$	$p = 0.267$	$p = 0.784$	$p = 0.962$	$p = 0.808$	$p = 0.751$	$p = 0.167$	$p = 0.439$	$p = 0.719$	$p = 0.703$	$p = 0.514$
Female, < 25 y (n = 28)	4.29 ± 0.76	3.64 ± 1.06	3.36 ± 0.87	3.71 ± 0.85	3.93 ± 0.81	3.96 ± 0.84	4.21 ± 0.79	3.11 ± 0.83	2.86 ± 0.71	3.07 ± 0.81	3.46 ± 0.64
Female, ≥ 25 y (n = 17)	4.35 ± 0.61	3.88 ± 0.86	3.53 ± 0.72	3.29 ± 1.16	3.53 ± 0.94	3.65 ± 1.06	3.94 ± 1.03	3.18 ± 0.88	2.82 ± 0.53	2.82 ± 0.53	3.18 ± 0.88
Male, < 25 y (n = 37)	4.51 ± 0.69	3.68 ± 1.08	3.24 ± 0.80	3.65 ± 0.86	3.84 ± 0.90	3.86 ± 0.98	3.70 ± 1.05	2.59 ± 0.98	2.59 ± 0.93	2.68 ± 1.20	3.43 ± 0.87
Male, ≥ 25 y (n = 19)	4.42 ± 0.69	3.42 ± 1.17	3.32 ± 1.11	3.21 ± 0.85	3.53 ± 0.77	3.42 ± 0.96	4.00 ± 1.05	2.37 ± 0.90	2.68 ± 0.95	2.58 ± 0.84	3.37 ± 0.90

*Significant (P ≤ 0.05). ***Significant (P ≤ 0.001).
